# Supplementary material for: Impacts drive lunar rockfalls over billions of years
Source: Nat Commun. 2020 Jun 8;11:2862. doi: 10.1038/s41467-020-16653-3 (PMC7280507; doi:10.1038/s41467-020-16653-3)
Supplement: Supplementary file 1 — Supplementary Information file [file 41467_2020_16653_MOESM1_ESM.pdf]

Supplementary Information for:

Impacts drive lunar rockfalls over billions of years

by Bickel et al. (2020)

Supplementary Figures

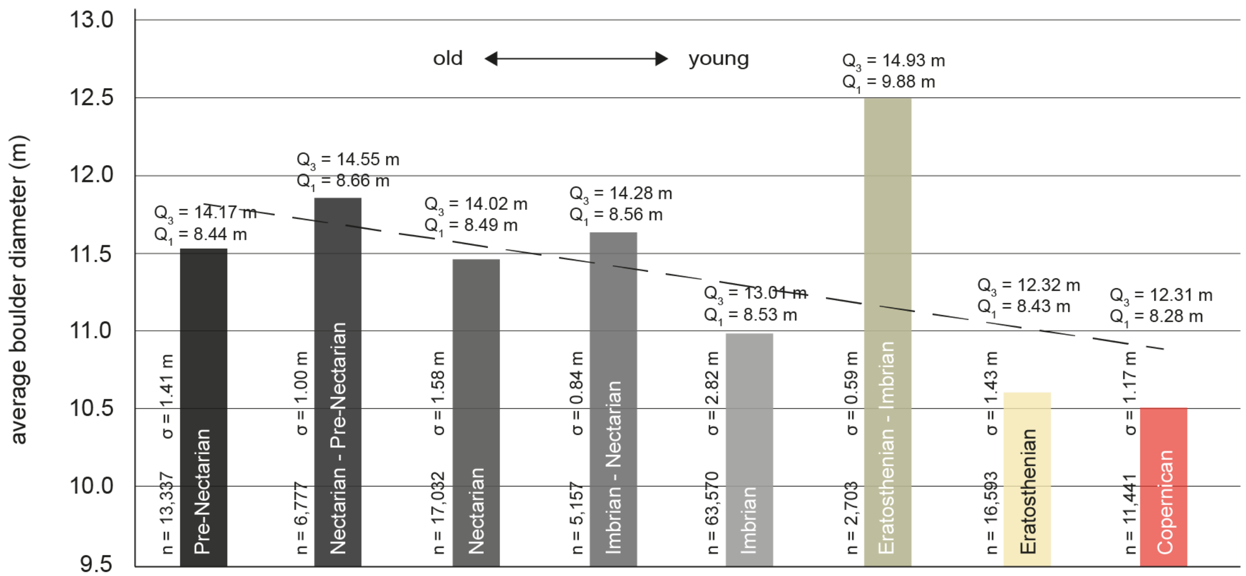

Supplementary Fig. 1: Average boulder diameter as function of terrane age. Rockfalls in older terranes show systematically larger average boulder diameters, as highlighted by the linear fit. The used number of rockfalls (n), the standard deviation (σ), and the first and third quartiles (Q_1 and Q_3) of each age class are shown.

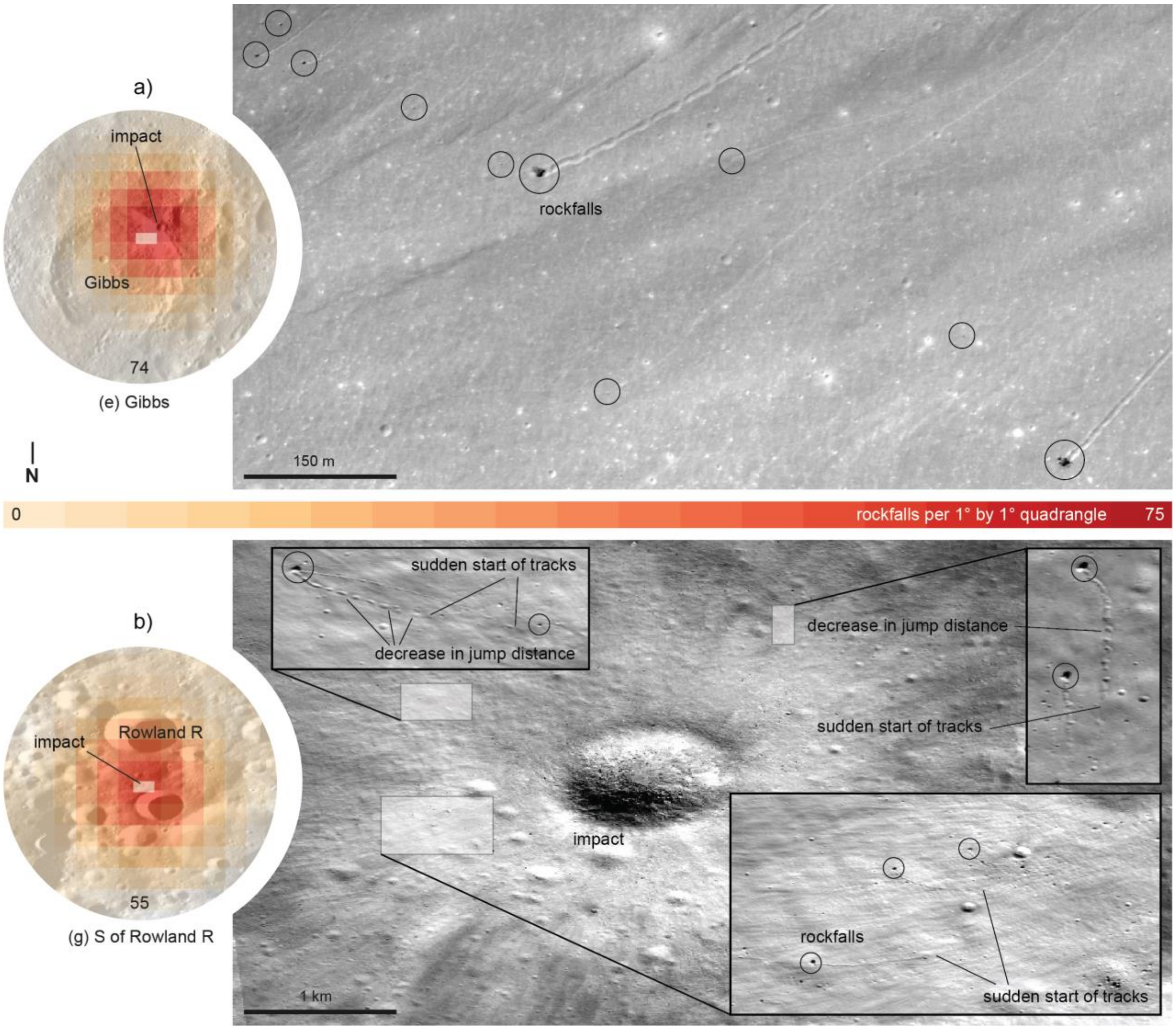

Supplementary Fig. 2: Examples of impact-induced rockfalls. a) rockfalls (circled) triggered by recent impact on Gibbs crater's NE slope. b) rockfalls triggered by recent impact south of Rowland R crater. The three insets show tracks with sudden starts as well as gradually decreasing boulder jump distances, indicating that the boulders have initially been airborne. In both examples, the rockfalls radiate away from the respective recent impact craters, further supporting the hypothesis of an impact-induced boulder displacement. The white rectangles in the heat map insets indicate the location of the two background NAC images. The peak count of spatial rockfall density is shown in the heat map insets per 1° by 1° quadrangle. Details of LRO NAC (LROC) images M185325951RC, M187007205LC, and M128041655LC. NAC Image credits to LROC/ASU/NASA.

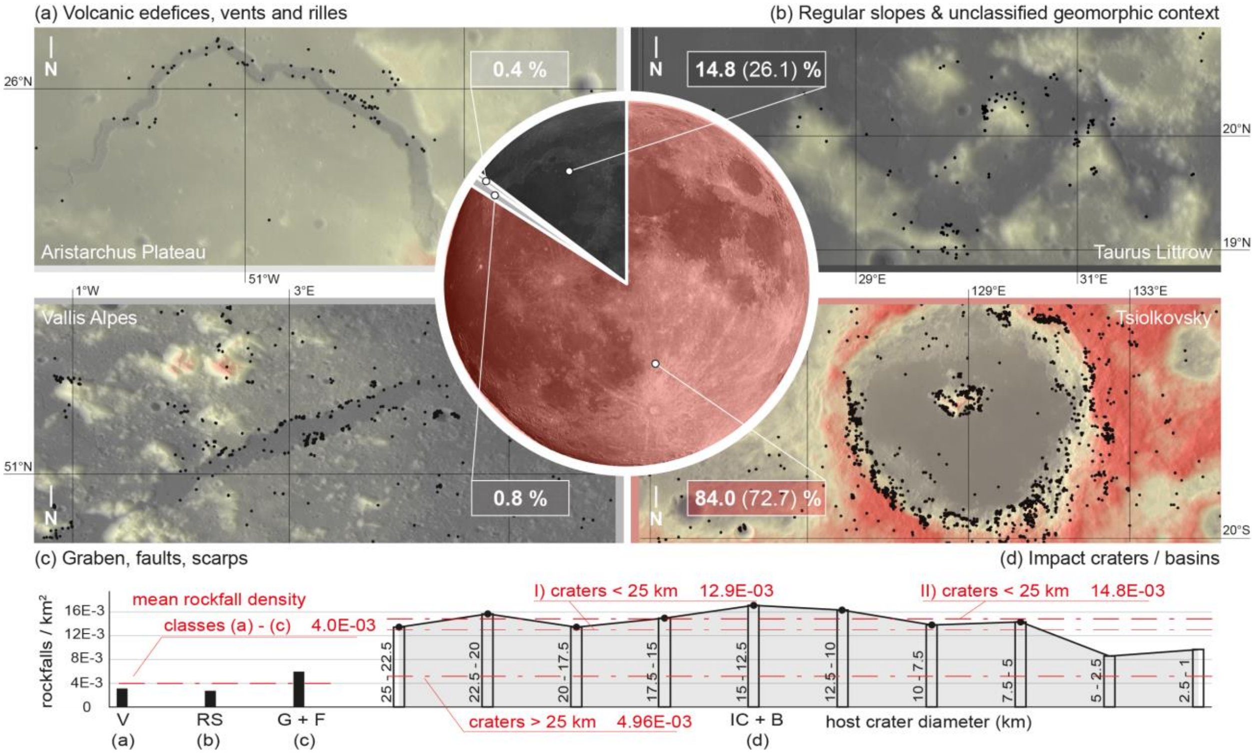

Supplementary Fig. 3: Rockfall counts throughout the different geomorphic contexts. Geomorphic settings that host lunar rockfall features: impacts / basins, graben, faults, scarps, volcanic edifices, vents and rilles, and regular slopes & unclassified geomorphic context. Black dots indicate detected rockfalls. The vast majority of recent lunar rockfalls occur in impacts, basins, and on associated central peaks (see d). The pie chart and the bold percentages indicate the rockfall count with considering craters between 1 and 5 km in diameter[1]; the numbers in brackets indicate the count without considering the 1 to 5 km crater population. The box plot shows the (mean) rockfall density per square kilometer for classes (a) through (c) and for craters with diameters between 1 and 25 km (in 10 bins) as well as the overall averages (red lines and numbers); I) denotes an average with the 1 to 5 km crater population, II) without. Craters < 25 km generally feature higher rockfall densities and craters with sizes between 5 and 25 km have similar rockfall densities, while craters smaller 5 km show slightly reduced densities. The diameter range between 1 and 25 km has been chosen as craters in this size range mostly consist of 'slope area' (i.e., no or a small, flat crater floor), whereas larger craters feature increasingly less 'slope area' but more 'flat area' in the center of the crater, potentially biasing the area normalization. Elevation model[2] and WAC mosaic in the background[3], red colors indicate high and black colors low elevations (in the map insets).

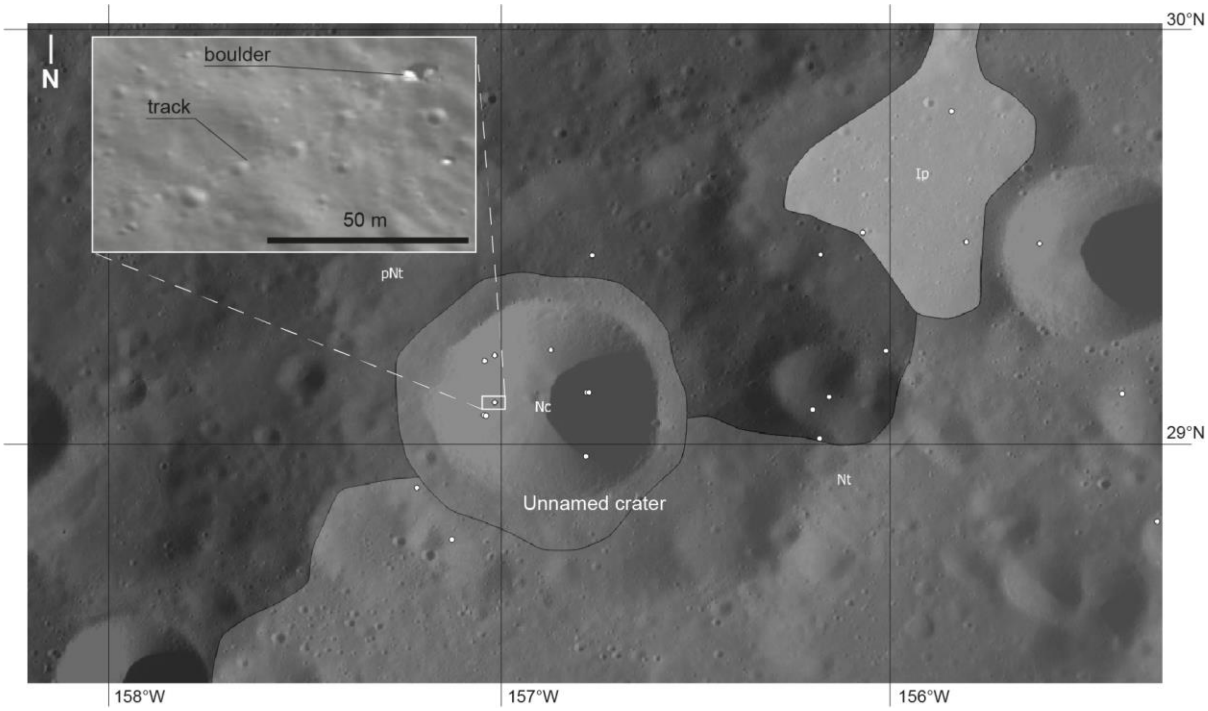

Supplementary Fig. 4: Example for rockfalls in ancient (Nectarian) terranes. Rockfalls (white shapes) in a Nectarian crater (Nc) prove that these ancient terranes have been subject to geologically recent erosion or are still being eroded today. Other rockfalls are located in surrounding Nectarian (Nt), pre-Nectarian (pNt), and Imbrian terranes (Ip). Inset shows a detail of one of the rockfalls (the boulder and its track) in the unnamed crater. WAC mosaic in the background[3], geological map taken from[4,5].

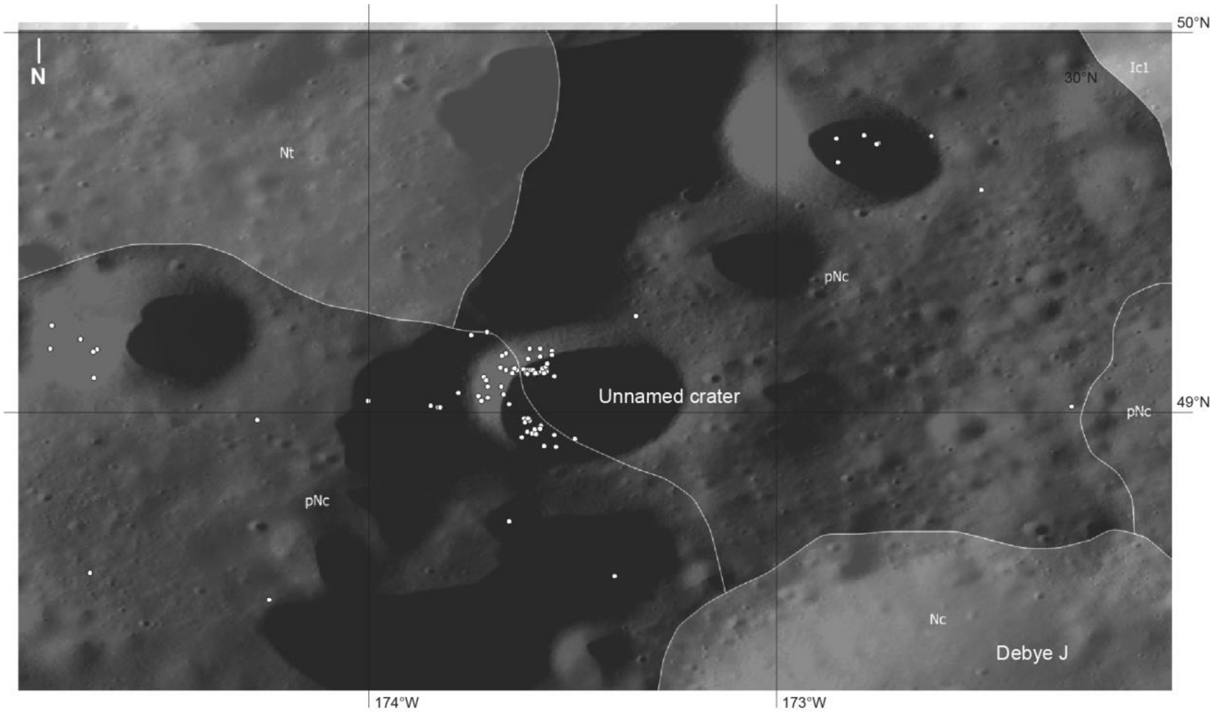

Supplementary Fig. 5: Example for potentially mis-classified (epoch-wise) rockfalls. This example illustrates the influence of potentially mis-classified (epoch-wise) craters: The rockfalls (white shapes) in the unnamed crater are categorized as pre-Nectarian (pNc) although the crater might actually be younger. The map appears not to consider this particular crater as a geologic contact runs right through it (white line). WAC mosaic in the background[3], geological map taken from[4,5].

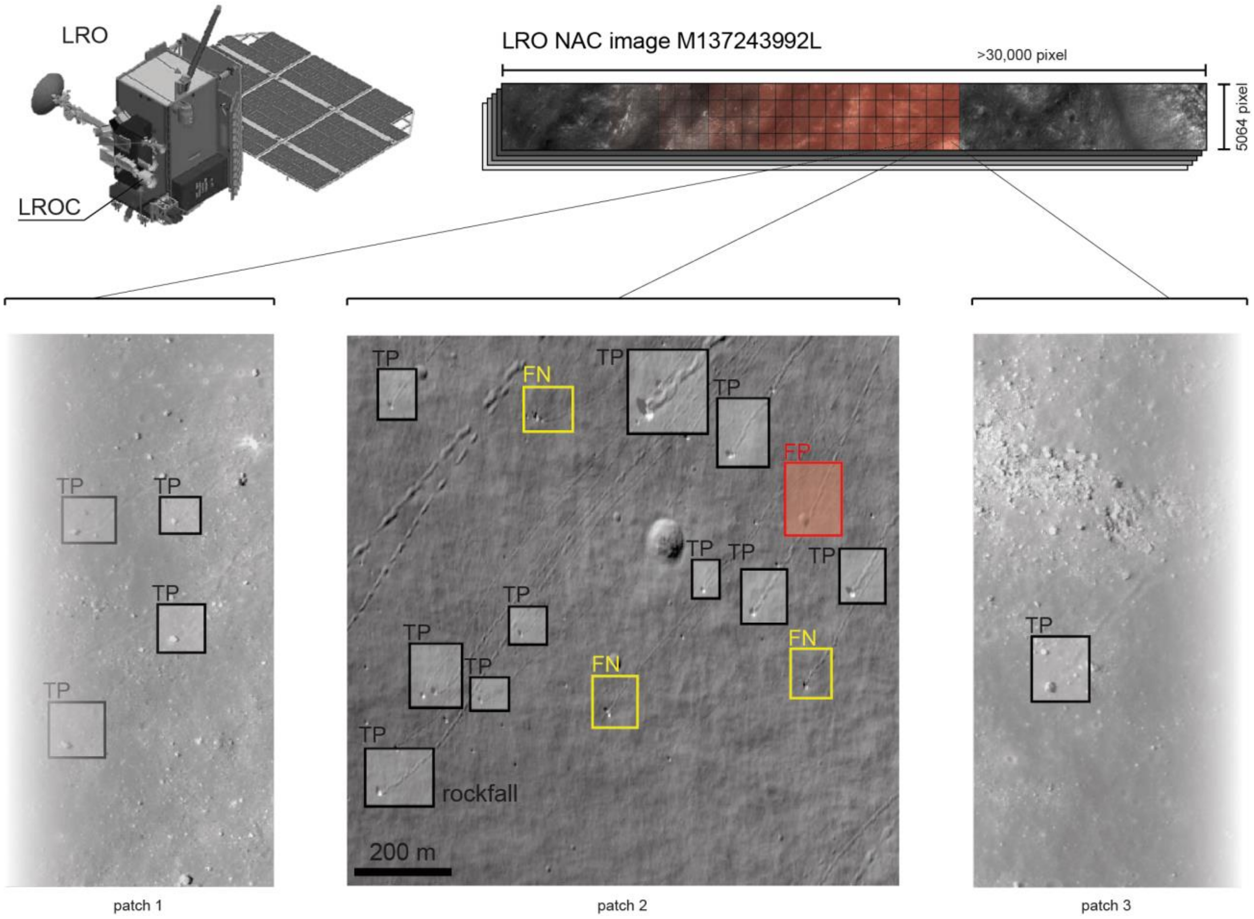

Supplementary Fig. 6: CNN-driven detection pipeline. Detections made by the CNN in LRO NAC image M137243992L taken over Atlas crater, lunar nearside. True positives (correct detections, TP, black), false positives (false detections, FP, red), and false negatives (missed detections, FN, yellow) are indicated. The diameter of the CNN-inferred bounding box can be used to estimate the diameter of the detected rockfalls[6]. Detections and bounding boxes have been visually enhanced (colors, infill, labels) to help illustrate the underlying concept. NAC Image credits to LROC/ASU/NASA.

Supplementary References

1. Robbins, S. J. (2018). A New Global Database of Lunar Impact Craters >1–2 km: 1. Crater Locations and Sizes, Comparisons With Published Databases, and Global Analysis. JGR: Planets, volume124, Issue4, pp. 871-892
2. Scholten, F.; Oberst, J.; Matz, K. D.; Roatsch, T.; Waehlisch, M.; Speyerer, E. J.; & Robinson, M. S. (2012). GLD100: The near-global lunar 100 m raster DTM from LROC WAC stereo image data. Journal of Geophysical Research: Planets, 117(E12). https://dx.doi.org/10.1029/2011JE003926
3. Wagner, R. V.; Speyerer, E. J; Robinson, M. S.; & LROC Team. (2015). New Mosaicked Data Products from the LROC Team. In Lunar and Planetary Science Conference (Vol. 46, abstract #1473). https://www.hou.usra.edu/meetings/lpsc2015/pdf/1473.pdf
4. USGS. (2013). Lunar 5M Geologic Map Renovation. URL: https://astrogeology.usgs.gov/search/map/Moon/Geology/Lunar_Geologic_GIS_Renovation_March2013 (Status: 11/04/19).

5. Fortezzo, C. M.; Hare, T. M. (2013). Completed digital renovation of the 1:5,000,000 lunar geologic map series. Lunar Plan. Sci. Conf., XLIV, abstract 2114.

6. Bickel, V. T.; Lanaras, C.; Manconi, A.; Loew, S.; Mall, U. (2018). Automated Detection of Lunar Rockfalls using a Convolutional Neural Network. IEEE Transactions on Geoscience and Remote Sensing, 57 (6), 3501-3511.
